# Effect of Lagoon and Sea Water Depth on *Gracilaria gracilis* Growth and Biochemical Composition in the Northeast of Tunisia

**DOI:** 10.1038/s41598-020-66003-y

**Published:** 2020-06-22

**Authors:** Fethi Mensi, Sarra Nasraoui, Saloua Bouguerra, Aziz Ben Ghedifa, Mohamed Chalghaf

**Affiliations:** 1 Institut National des Sciences et Technologies de la Mer, B3 Aqua laboratory, Centre de recherche de Kheiredine, 29 Rue Général Kheiredine, 2015 Le Kram, Tunisie; 2 Institut National Agronomique de Tunisie 43, Avenue Charles Nicolle 1082 -Tunis, Mahrajène, Tunisie; 3 Facultés des Sciences Mathématiques physiques et Naturelles Campus Universitaire El-Manar, 2092 El Manar Tunis, Tunisie; 4 Institut supérieur de Pèche et d’Aquaculture de Bizerte, BP 15 Errimel, 7080 Menzel Jemil Bizerte, Tunisie

**Keywords:** Biochemistry, Ocean sciences

## Abstract

This study evaluated the growth and biochemical composition of farming *Gracilaria gracilis* (Stackhouse) M. Steentoft, L. M. Irvine & W. F. Farnham in the Bizerte Lagoon (BL) and Bizerte Bay (BB) in the North Coast of Tunisia, using lantern nets. Effects of site and depth on alga daily growth rate (DGR) and biochemical composition were investigated. The DGR was affected by culture site (1.42 ± 0.65% day^−1^ and 1.19 ± 0.34% day^−1^ for the BL and the BB respectively). Agar yield, was higher (*p* < 0.05) in the BB than the BL (23.31 ± 2.64% vs. 19.19 ± 2.32%) with a higher (*p* < 0.05) 3,6-anhydrogalactose (3,6-AG) contents (41.37 ± 3.68% vs 23.30 ± 5.40%) and a lower (*p* < 0.05) sulphate degree (6 ± 2.00% vs 8.80 ± 0.86%). The proteins contents were independent of the site and depth of culture (20.74 ± 7.22% and 22.02 ± 6.34% for the BL and the BB respectively). R-phycoerythrin (R-PE) contents were significantly higher (*p* < 0.05) in the BB (0.86 ± 0.31 mg g^−1^) than those obtained in the BL (0.33 ± 0.12 mg g^−1^). The salinity, transparency, nitrate and ammonium were monitored in both sites, and their influences were discussed. Our results suggest that *G. gracilis* cultured in Bizerte Bay can be used in a cascading biorefinery approach.

## Introduction

Global seaweed production, largely derived from aquaculture (96.5 percent by volume of the wild-collected and cultivated aquatic plants combined), has changed considerably since 2005^1^. This production increased from 13 million in 2005 to reach 30 million tons (live weight) in 2016. Moreover, 99% of the world’s production comes from Asian countries, notably China (47.9%), Indonesia (38.7%), Philippines (4.7%), Republic of Korea (4.5%), Democratic People’s Republic of Korea (1.6%), Japan (1.3%) and Malaysia (0.7%). Furthermore, trade in aquatic plants increased from USD 60 million in 1976 to more than USD 1 billion in 2016, with Indonesia, Chile and the Republic of Korea the major exporters, and China, Japan and the United States of America the leading importers. Accordingly, red seaweed production accounts for 53% of the world production^1^. The most exploited red algae, expressed in a million tons year^−1^, are *Euchema* seaweeds nei and *Eucheuma* spp. (10.5), *Gracilaria* spp. (4.1) and *Porphyra* spp. (1.3). In contrast, the red alga *Gracilaria* spp. was barely farmed at all in 1990. However, the increase in *Gracilaria* spp. production, by aquaculture, is mainly due to the growing demand for agar. Consequently, *Gracilaria* spp. aquaculture has been initiated by many countries, in different regions of the world, such as Thailand, Chile, Vietnam, Portugal, Australia, Brazil and India^2^.

The red alga *Gracilaria gracilis*, which grow in Asian coasts, was introduced into the Mediterranean Sea^3^, and established in the lagoons^4–7^. Consequently, the alga was found all year round, but is the most component of the BL flora between April and June^4^. In Tunisia, the quantity of seaweeds harvested, was restricted along the Bizerte Lagoon and the Tunis Lake, which is inadequate to supply the raw material requirement of the industries. Hence, *G. gracilis* aquaculture was indicated as the main solution^4^. However, the BL was chosen to initiate many cultivation attempts of *G. gracilis*. As a result, a low biomass supply was obtained due to the interaction with environmental factors (Grazing, epiphitism, hydrodynamism, etc…) and limited surface suitable for the benthic culture methods. As a consequence, these culture methods were applied only in a depth less than 2 m, which represent a 10% of the BL surface^8^. Furthermore, agar obtained (gel strength less than 400 g cm^−2^) was of low quality compared to that extracted from *Gelidium latifolium* (gel strength higher than 800 g cm^−2^), which considered as the highest and used in many industrial applications^9^. Hence, no large-scale culture of *G. gracilis* is being developed outside the Asian region. That’s why suspended culture method should be an alternative to enhance biomass production in lagoon and sea^8,9^. In addition, when we move toward the lagoon depth and sea, we will look for the possibilities to improve the alga quality.

Around the world *Gracilaria* biomass was used usually for agar extraction. In contrast, it contains a wide variety of valuable compounds, such as proteins and pigments^10^. The cascading biorefinery, as an alternative to single product extraction approach, aim to extract all components present in the algae biomass^11,12^. According to^13,14^, it is financially attractive to firstly extract R-PE, and then agar will be extracted from the by-product obtained. Furthermore, the agar by-product can be treated as waste to produce biofuels. Consequently, it is necessary to study the ecophysiology responses of *G. gracilis* to the various factors, which affect their growth and chemical composition, such as light intensity, nutrients, salinity and temperature variations. In Tunisia, no attempt has been done to understand *G. gracilis* behavior in deeper lagoon and sea. Therefore, the purpose of this study was to provide new insights on *G. gracilis* growth capacities and biochemical composition (agar, proteins and R-PE) in the BL and the BB. In addition, it may also provide knowledge on algal biomass uses.

## Materials and methods

### Preparation of experimental material

**Figure 1:**
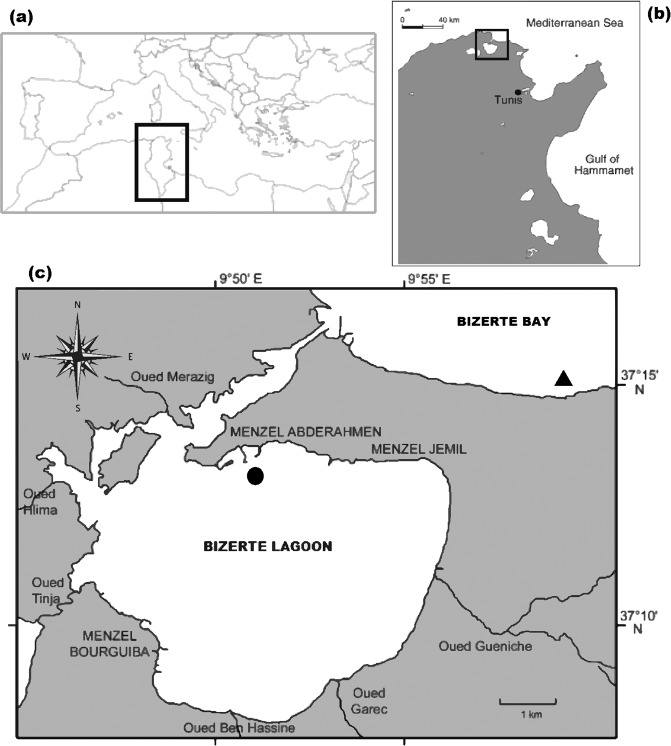
(**a**) Situation on Tunisia map within the Mediterranean region. (**b**) *Gracilaria gracilis* culture area. (**c**) Culture site location in the Bizerte Lagoon and Bay.

*Gracilaria gracilis* was collected between 0.5 and one meter depth from the Bizerte Lagoon (Fig. 1), North Tunisia (37°13′N; 9°55′E) at the end of Februray 2016. The collected thalli were transported to the laboratory in a cool environment to reduce stress. Collected samples were subjected to a series of washing steps using filtered sea water to eliminate diatoms contamination, epiphytes and other competing organisms. Then, the cleaned thalli, were placed in a tank with physicochemical parameters (dissolved oxygen, temperature and salinity) similar to those of the lagoon. Homogenous thalli with bright-red color, similar lengths and branches were selected.

### Cultivation of gracilaria gracilis

**Figure 2:**
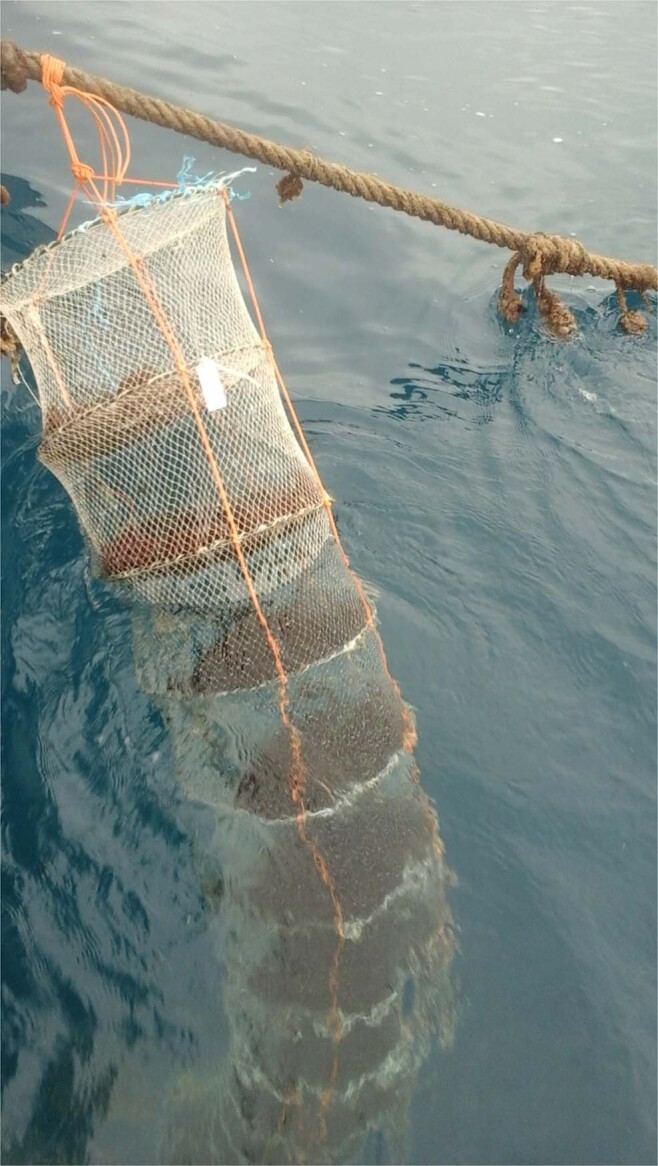
Lantern nets hang down into the water column in Bizerte Bay.

The *G. gracilis* culture experiments were conducted from March to May 2016 (90 days), in the Bizerte Lagoon (BL; 37°13N, 9°51′W) and Bay (BB; 37°15N, 9°59 W) in North Tunisia (Fig. 1). The experimental sites were located within sites specifically devoted to aquaculture with average water depths of 8 and 20 m for the BL and the BB, respectively. The suspended culture in the two sites was carried out using lantern nets (Fig. 2). The lantern net is formed by solid steel rings and crossbars coated with anti-corrosive. The crossbars were used to fix the *G. gracilis* thalli. Each lantern net is 3.75 m in length and contained fifteen experimental baskets (40 cm of diameter and 0.25 m in height) enclosed by monofilament netting of 6 mm mesh size. Each compartment has an opening through which thalli can be inserted or removed. Lantern nets loaded with thalli were hung down into the water column from long lines with buoys, which were placed in 1.3 m intervals. Lantern nets followed the tide without changing their position relative to the water surface during tidal cycles; get a vertical suspension due to a heavy-duty chain length. A synthetic rope of 36 mm in thickness and threefold the water depth in length was attached to the chain and rose to the marker buoys. Prepared tufts (500 g) were placed in the basket, being attached to the crossbars by a braid wire. Every lantern net, as well as baskets, was marked in such a way that each tuft represents an experimental unit. Three lantern nets were used in each site. As a result, at each depth, there were three experimental samples for statistical analyses. Lantern nets were then immersed in the filtered sea water, whose salinity was gradually increased into that of the BB to prevent thallus loss until they were transferred to the culture site.

Weights of tufts were recorded at the beginning and at the end of the experiments to quantify algae growth. DGR was calculated by the formula in^15^:

1 $${\rm{DGR}}( \% \,{{\rm{day}}}^{-1})={\rm{In}}({\rm{Wf}}/{\rm{W}}0)/{\rm{t}}\times 100$$

where Wf is the final fresh weight after the t days of culture, and W0 is the initial fresh weight.

At the end of the culture period, a percentage tufts loss (PTL) was calculated using the following formula:

2 $${\rm{PTL}}( \% )=({\rm{Nf}}/{\rm{N}}0)\times 100$$

where Nf and N0 were the final and initial number of tufts.

### Environmental measures

Three water samples were collected every month (March, April and May) from the BL and the BB, in acid-washed 1 L plastic bottles from a depth of 0.5 to 1 m using a Van Dorn water sampler three liters. For each sample taken, three replicates were analysed. Water samples fixed with 0.5 mL of H_2_SO_4_ 4N. In the laboratory, triplicate samples (3 L) were filtered using Whatman® Grade GF/C Glass Microfiber filters to determine NO_3_^−^, NH_4_^+^ and PO_4_^−^. Water samples were analysed for Ν and Ρ according to^16,17^ methods. During the culture period Secchi depth, temperature, salinity and dissolved oxygen were measured. Three measures of Secchi depth per month were done according to the recommendations of^18^, by the same person at the same time of the day. Water temperature, oxygen and salinity were measured *in situ* using Hack multi-parameter (HQ40D), at the same time in the day, according to the recommendations of^19^.

### Agar extraction and quality determination

At the end of the culture period, dry-cleaned seaweed samples were first washed with tap water to remove salts. Then, they were placed in 400 mL of a 5% H_2_SO_4_ solution for 1 h at room temperature and further rinsed thoroughly using tap water. Agar extraction was performed in acid-washed 500 mL glass bottles at 100 °C for 90 min using a 2.5 (% w/v) of dried alga and distilled water. The heated solution was then filtrated using a bûchner filter and a vacuum pump. The filtrate obtained was then transferred to a flat steeled recipient until it was cooled at room temperature for 20 min and then frozen overnight at −18 °C. Next day, the filtrate was thawed at room temperature until a thin agar film was formed. The agar yield was calculated as follows:

3 $${\rm{Agar}}\,{\rm{yield}}( \% )=({\rm{Wa}}/{\rm{Ws}})\times 100$$

where Wa is the dry agar weight and Ws is the dry seaweed weight (g).

Sulfaphate content in polysaccharides was determined turbidimetrically using Barium chloride after acid hydrolysis as described by^20^. First, a barium chloride-gelatin solution was prepared by dissolving 600 mg of gelatin in 200 mL heated osmosis water (60–70 °C) and placed for 1 to 2 h at room temperature after cooling to 4 °C during 16 h. Then, 2 g of Barium chloride was dissolved in the resulting gelatin solution and left to stand for 2–3 h. Next, a hydrochloric acid solution (HCL 0.5N) was prepared. Thereafter, a sulfate standard curve was generated from a series of K_2_SO_4_ solutions (3 mg mL^−1^) containing between 0.02 and 0.20 mg mL^−1^. Lastly, agar (10 mg) was weighed and dissolved in 0.5 mL of hydrochloric acid for two hours at 100 °C. The hydrolyzate was compensated to 10 mL and centrifuged at 5000 G for 10 minutes. Then, 1 mL was placed in a test tube with the presence of 1 mL of HCl (0.5N) and 0.5 mL of barium chloride-gelatin solution, then compensated by 9 mL of osmosis water and mixed by vortex. After 15 min at room temperature, the absorbance was determined at λ = 360 nm with UV-visible spectrophotometer (Jenway 6405 type) and the absorbance value was obtained. Hydrochloric acid solution was used as a blank.

The 3,6-AG content was determined by the colorimetric method of^21^, using the resorcinol–acetal reagent and fructose as standard. The acetal solution was prepared by diluting 1 mL of acetal in 100 mL of deionized water. The resorcinol solution was prepared by diluting 150 mg of resorcinol in 100 mL of deionized water. Then, 9 mL of resorcinol solution was added to 1 mL of acetal solution diluted to 1/25. Next, 2 mL of agar solution (0.02 mg mL^−1^) was transferred into a test tube containing 10 mL of cold resorcinol-acetal reagent, mixed thoroughly and allowed to cool in ice bath for 3 min. Then, the tube was placed into water bath (80 °C) for 10 min. After cooling (15 mn) at room temperature, the absorbance was determined at λ = 555 nm with UV-visible spectrophotometer (Jenway 6405 type). A standard curve was prepared using D-fructose solution concentration, ranging from 0.018 to 0.09 mM. The 3,6-AG content was calculated and expressed as the percentage (dry weight basis). Experiments were performed in triplicate.

### Determination of crude protein and R-PE

Crude protein was determined according to the^22^ method. Fresh alga (1 g) was placed in 20 mL of deionized water. A seaweed extract sample (1 mL) was putted in a hemolytic tube to which was added 2 mL of Coomassie Blue reagent and then homogenized. Absorbance was determined at λ = 595 nm after 5 min, using an UV-visible spectrophotometer (Jenway 6405 type). Curve calibration was performed using a Bovine Serum Albumin (BSA) solution between 0 and 2.0 mg mL^−1^. Protein content Q (mg) in seaweed samples was calculated as follows:

4 $${\rm{Q}}={\rm{V}}\ast {\rm{C}}$$

where C is the protein concentration (mg g^−1^ fresh weight), obtained using the calibration curve; V = initial sampling volume (mL). Results are presented as percentage of dry weight (15%). The R-PE content was determined as described by^13^ and the absorbance was measured at 565 nm, which is the maximum of R-PE absorbance. Beer-Lambert law established the absorbance at 565 nm as follows:

5 $${\rm{A}}={\rm{\varepsilon }}\ast {\rm{L}}\ast {{\rm{C}}}_{1}=({\rm{\varepsilon }}\ast {\rm{L}}\ast {{\rm{C}}}_{2})/({\rm{MV}})$$

where C_2_ was calculated by the following equation:

6 $${{\rm{C}}}_{2}={\rm{A}}\ast \frac{260000}{2000000}=0.13\ast {\rm{A}}$$

A: absorbance at 565 nm,

ε: R-PE extinction coefficient (2.10^6^ M^−1^ cm^−1^),

L: optic length (=1 cm),

C_1_: molar concentration of R-PE (M),

C_2_: Concentration of R-PE (mg mL^−1^),

MW: molecular weight of R-PE (260000 Da).

### Statistical analyses

All results were expressed as mean ± Standard deviation. The Daily growth rate (% day^−1^), Agar yields (%), sulphate (%), 3,6-AG (%), protein (%) and R-PE (mg g^−1^ fw) obtained in different depths and sites were examined using the statistical package Statistica, version 5.1^23^, as shown in Table 1. After verification of the homogeneity of the variances and the normality of the data, the results, were subjected to two-way ANOVA analysis to assess the impacts of sites and depths according to the GLM procedure. When ANOVA proved to be significant, the Duncan’s test was used to compare averages; the significance level of 5% was retained.

**Table 1:** Physico-chemical parameters at the Bizerte Lagoon and Bay water. Values are means ± SD; n = 9 for physical parameters and n = 18 for chemical parameters. Means in same row followed by same superscript letter are not significantly different according to the Duncn’s test (P > 0.05).

	BL	BB
**Chemical parametres**		
Nitrate (μ mol L^−1^)	21.87^a^ ± 2.17	1.64^b^ ± 0.59
Ammonium (μ mol L^−1^)	13.32^a^ ± 2.80	11.73^a^ ± 1.91
Orthophosphate (μ mol L^−1^)	4.53^a^ ± 0.49	3.54^a^ ± 0.71
**Physical parametres**		
Secchi depth (m)	2.37^a^ ± 0.25	4.50^b^ ± 0.50
Salinity (psu)	35.86^a^ ± 0.04	36.90^b^ ± 0.36
Temperature (°C)	17.45^a^ ± 3.57	19.43^a^ ± 1.25
Dissolved oxygen (mgL^−1^)	7.98^a^ ± 0.02	8.28^a^ ± 0.71
pH	8.18^a^ ± 0.07	8.36^a^ ± 0.16

BL: the Bizerte lagoon; BB: the Bizerte bay; Means in same row followed by same superscript letter are not significantly different (P > 0.05).

### Ethical statement

This article doesn’t contain any studies with animals performed by any of the authors.

## Results

The physicochemical characteristics of water in the BL and the BB, during the culture period, are shown in Table 1. Consequently, the water transparency (m), salinity (psu) and nitrate concentration (µmol L^−1^) varied significantly between the BL and the BB. In contrast, the water temperature (°C), dissolved oxygen (mgL^−1^), pH, ammonium (µmol L^−1^) and orthophosphate concentration (µmol L^−1^) were similar. The BL water was less transparent compared to the BB (2.37 ± 0.25 m vs 4.50 ± 0.50 m). The average salinity were different between the BL and the BB (35.86 ± 0.04 psu vs 36.90 ± 0.36 psu). The nitrate showed the higher concentrations in the BL compared to the BB (21.87 ± 2.17 μmol L^−1^ vs 1.64 ± 0.59 μmol L^−1^).

Results of DGR (%), agar yields (%),3,6-AG (%), sulphate (%), proteins (%) and R-PE (mg g^−1^ fresh wt) were shown in Tables 2, 3, 4 and Supplementary Fig. S1. Hence, *G. gracilis* grows differently (P < 0.05) between the site and the culture depth (Table 2). Accordingly, the DGR recorded in the BL was higher than that of the BB (1.42 ± 0.65% day^−1^ vs 1.19 ± 0.34% day^−1^). In the BL, we recorded a decrease of DGR from the surface to 3.75 m. Consequently, a highest DGR were recorded between the surface and 1 m. As a result, DGR recorded at 1 m is the highest (2.33 ± 0.03% day^−1^). Whereas, a lowest DGR ( <1%) was recorded over 3 m. In addition, the DGR at 3.5 m does not exceed 0.5% day^−1^ and showed null values at 3.75 m, by far the lowest, due to thallus degeneration. In contrast, DGR recorded in the BB were homogenous in the studying depths (p > 0.05) and varied from 0.86 ± 0.32% day^−1^ to 1.57 ± 0.42% day^−1^ (Table 3). In the BL, the thalli loss was insignificant from the water surface to 1.5 m. Consequently, the PTL ranges between 1% and 2%. In contrast, in the BB, up to 1.5 m, we recorded the higher PTL, especially near the water surface (10%).

**Table 2:** Daily Growth Rate (DGR %), Agar yield (%), Sulfate (%), 3,6-Anhydrogalactose (3,6-AG %), Proteins **(%)** and R-phycoerythrin **(**R-PE mg g^−1^fw) obtained from *Gracilaria gracilis* samples cultured at different depths in two sites (Bizerte Lagoon and Bizerte Bay, Northeast Tunisia) analyzed by ANOVA at 95% confidence level.

Factors	*SS*	*df*	*MS*	*F*	*p*
**DGR (%)**					
Intercept	154.07	1	154.07	1557.88	<0.05
Site	1.22	1	1.22	12.36	<0.05
Depth	7.85	14	0.56	5.67	<0.05
Site*Depth	10.24	14	0.73	7.39	<0.05
Error	5.93	60	0.10		
**Agar (%)**					
Intercept	40636.38	1	40636.38	6314.463	<0.05
Site	382.75	1	382.75	59.475	<0.05
Depth	110.93	14	7.92	1.231	>0.05
Site*Depth	58.34	14	4.17	0.648	>0.05
Error	386.13	60	6.44		
**Sulfate (%)**					
Intercept	4932.622	1	4932.622	3507.418	<0.05
Site	176.084	1	176.084	125.207	<0.05
Depth	54.125	14	3.866	2.749	<0.05
Site*Depth	74.098	14	5.293	3.763	<0.05
Error	84.380	60	1.406		
**3,6-AG(%)**					
Intercept	97159.40	1	97159.40	36228.44	<0.05
Site	6518.91	1	6518.91	2430.75	<0.05
Depth	853.10	14	60.94	22.72	<0.05
Site*Depth	1196.31	14	85.45	31.86	<0.05
Error	160.91	60	2.68		
**Proteins(%)**					
Intercept	41150.41	1	41150.41	979.3834	<0.05
Site	36.62	1	36.62	0.8715	>0.05
Depth	976.25	14	69.73	1.6596	>0.05
Site*Depth	661.06	14	47.22	1.1238	>0.05
Error	2521.00	60	42.02		
**R-PE(mgg**^**−1**^**fw)**					
Intercept	31.92	1	31.92	535.76	<0.05
Site	6.30	1	6.30	105.83	<0.05
Depth	1.15	14	0.08	1.38	>0.05
Site*Depth	0.23	14	0.02	0.27	>0.05
Error	3.57	60	0.06

**Table 3:** Daily growth rate and proximate biochemical composition of *Gracilaria gracilis* cultivated in Bizerte lagoon (BL) and bay (BB) at different depth (from 0.25 m to 3.75 m). Values are means ± SD; n = 9. Means with the same superscripts are not significantly different (*p* = 0.05).

	Depth (m)	DGR (%)	Agar (%)	Sulfate (%)	3,6-AG(%)	Proteins (%)	R-PE (mgg^−1^fw)
BL	0.25	2.33^a^ ± 0.03	18.40^a^ ± 1.44	7.97^e^ ± 0.54	32.41^a^ ± 1.39	22.06^a^ ± 10.72	0.25^a^ ± 0.05
BL	0.50	1.96^ab^ ± 0.46	18.07^a^ ± 2.25	8.70^bcde^ ± 0.83	32.79^a^ ± 1.81	17.10^a^ ± 6.96	0.24^a^ ± 0.04
BL	0.75	2.06^ab^ ± 0.32	20.70^a^ ± 0.26	8.43^cde^ ± 0.54	32.98^a^ ± 2.30	20.13^a^ ± 7.76	0.25^a^ ± 0.03
BL	1.00	1.96^ab^ ± 0.19	19.43^a^ ± 2.63	8.13^e^ ± 0.40	40.68^a^ ± 3.30	16.36^a^ ± 6.58	0.31^a^ ± 0.09
BL	1.25	1.75^abc^ ± 0.27	18.90^a^ ± 1.65	8.28^de^ ± 0.29	29.98^a^ ± 0.57	18.50^a^ ± 7.42	0.28^a^ ± 0.03
BL	1.50	1.58^bc^ ± 0.13	21.23^a^ ± 3.96	8.39^cde^ ± 0.17	31.47^a^ ± 1.34	16.56^a^ ± 6.30	0.31^a^ ± 0.06
**BL**	1.75	1.34^bc^ ± 0.39	17.43^a^ ± 2.67	9.42^abcd^ ± 0.24	32.64^a^ ± 1.15	21.86^a^ ± 9.56	0.36^a^ ± 0.16
BL	2.00	1.49^bc^ ± 0.27	19.27^a^ ± 1.36	9.58^abc^ ± 0.17	31.93^a^ ± 1.86	21.77^a^ ± 8.46	0.37^a^ ± 0.19
BL	2.25	1.42^bc^ ± 0.11	21.57^a^ ± 3.71	9.42^abcd^ ± 0.29	30.42^a^ ± 1.10	22.64^a^ ± 10.59	0.37^a^ ± 0.17
BL	2.50	1.53^bc^ ± 0.14	18.50^a^ ± 2.11	7.52^e^ ± 0.52	35.97^a^ ± 5.95	23.97^a^ ± 8.64	0.41^a^ ± 0.19
BL	2.75	1.43^bc^ ± 0.16	19.17^a^ ± 2.10	9.69^ab^ ± 0.46	36.43^a^ ± 0.63	29.63^a^ ± 8.23	0.43^a^ ± 0.21
BL	3.00	1.05^c^ ± 0.15	18.00^a^ ± 2.55	8.62^bcde^ ± 0.40	30.73^a^ ± 0.46	27.18^a^ ± 6.52	0.45^a^ ± 0.19
BL	3.25	1.05^cd^ ± 0.07	18.80^a^ ± 4.16	9.96^a^ ± 0.50	35.04^a^ ± 1.84	26.05^a^ ± 2.79	0.30^a^ ± 0.03
BL	3.50	0.57^d^ ± 0.25	19.07^a^ ± 0.95	7.97^e^ ± 0.37	27.26^a^ ± 0.53	22.30^a^ ± 3.09	0.32^a^ ± 0.05
BL	3.75	−0.16^e^ ± 0.61	19.27^a^ ± 1.82	9.96^a^ ± 0.70	32.12^a^ ± 1.52	24.20^a^ ± 4.05	0.32^a^ ± 0.05
BB	0.25	0.86^a^ ± 0.32	21.13^a^ ± 1.44	7.74^ab^ ± 2.60	45.82^abc^ ± 1.27	15.38^a^ ± 5.69	0.63^a^ ± 0.13
BB	0.50	1.03^a^ ± 0.48	24.03^a^ ± 2.25	5.38^ab^ ± 0.75	46.12^ac^ ± 2.78	15.47^a^ ± 3.85	0.64^a^ ± 0.11
BB	0.75	0.99^a^ ± 0.43	22.47^a^ ± 0.26	7.40^ab^ ± 2.38	40.78^bcd^ ± 0.81	27.74^a^ ± 0.47	0.65^a^ ± 0.09
BB	1.00	0.91^a^ ± 0.78	20.87^a^ ± 2.63	5.91^ab^ ± 0.46	41.31^abcd^ ± 0.41	16.30^a^ ± 4.70	0.73^a^ ± 0.22
BB	1.25	1.57^a^ ± 0.42	22.73^a^ ± 1.65	5.42^ab^ ± 1.37	39.94^bd^ ± 1.51	15.45^a^ ± 2.76	0.77^a^ ± 0.09
BB	1.50	1.32^a^ ± 0.39	23.40^a^ ± 3.96	8.28^a^ ± 1.85	40.05^d^ ± 1.93	25.39^a^ ± 6.42	0.81^a^ ± 0.15
**BB**	1.75	1.27^a^ ± 0.15	21.27^a^ ± 2.67	7.90^ab^ ± 1.79	46.96^a^ ± 4.52	18.14^a^ ± 3.04	0.82^a^ ± 0.41
BB	2.00	1.50^a^ ± 0.29	25.23^a^ ± 1.36	4.58^ab^ ± 0.71	39.83^bcd^ ± 0.30	19.23^a^ ± 6.00	0.82^a^ ± 0.49
BB	2.25	1.38^a^ ± 0.07	25.50^a^ ± 3.71	5.84^ab^ ± 2.89	37.96^d^ ± 0.35	28.88^a^ ± 10.19	0.82^a^ ± 0.43
BB	2.50	1.23^a^ ± 0.01	22.47^a^ ± 2.11	5.30^ab^ ± 0.86	42.19^abcd^ ± 2.94	17.06^a^ ± 1.09	0.93^a^ ± 0.50
BB	2.75	1.18^a^ ± 0.11	22.93^a^ ± 2.10	8.39^b^ ± 0.29	45.74^ac^ ± 1.52	20.87^a^ ± 5.09	0.96^a^ ± 0.56
BB	3.00	1.17^a^ ± 0.21	23.60^a^ ± 2.55	4.84^ab^ ± 1.32	36.74^d^ ± 2.29	23.44^a^ ± 4.41	0.97^a^ ± 0.49
BB	3.25	1.18^a^ ± 0.22	22.90^a^ ± 4.16	3.40^b^ ± 1.04	38.61^d^ ± 5.02	18.61^a^ ± 2.91	1.07^a^ ± 0.07
BB	3.50	1.09^a^ ± 0.23	24.90^a^ ± 0.95	5.19^ab^ ± 1.94	39.41^d^ ± 0.37	20.86^a^ ± 7.37	1.12^a^ ± 0.13
BB	3.75	1.19^a^ ± 0.24	26.23^a^ ± 1.82	4.50^ab^ ± 1.00	39.06^d^ ± 1.44	28.38^a^ ± 5.29	1.16^a^ ± 0.12

BL: the Bizerte Lagoon; BB:the Bizerte Bay; DGR: Daily Growth Rate;3,6-AG(%):3,6-anhydrogalactose; R-PE: R-phycoerythrin; SD: Standard deviation.

**Table 4:** Daily growth rate and proximate biochemical composition of *Gracilaria gracilis* cultivated in Bizerte Lagoon and Bizerte Bay. Values are means ± SD of results obtained at different depth (from 0.25 m to 3.75 m); n = 45. Means in same row followed by same superscript letter are not significantly different according to the Duncn’s test (P > 0.05).

	BL	BB
DGR(%)	1.42^a^ ± 0.65	1.19^b^ ± 0.34
Agar(%)	19.19^b^ ± 2.32	23.31^a^ ± 2.64
Sulfate(%)	8.80^a^ ± 0.86	6.00^b^ ± 2.00
3,6-AG(%)	23.30^b^ ± 5.40	41.36^a^ ± 3.68
Proteins(%)	20.74^a^ ± 7.22	22.02^a^ ± 6.34
R-PE(mgg^−1^fw)	0.33^b^ ± 0.12	0.86^a^ ± 0.31

BL: the Bizerte Lagoon; BB: the Bizerte Bay; DGR: Daily Growth Rate;3,6-AG(%):3,6-anhydrogalactose; R-PE: R-phycoerythrin; SD: Standard deviation.

The agar yields (Tables 3 and 4), which depend (p < 0.05) on the site of production, were 19.19 ± 2.32% and 23.31 ± 2.64% for the BL and the BB respectively. Inversely, the effect of the culture depth was not significant (p > 0,05) and homogeneous distribution in the whole studying water column was recorded. The 3,6-AG content and sulphate degree of agar varied significantly between the sites and the depths (p < 0.05). In the BB, the 3,6-AG content of agar was twice as high as that obtained in the BL (41.37 ± 3.68% vs 23.30 ± 5.40%) (Table 4). In addition, in the BL, the highest 3,6-AG content was recorded at 3.25 m, the lowest at 3.50 m and an irregular distribution between the other depths (Table 3). However, in the BB the highest amount of 3,6-AG (> 45%) was found near the water surface (<0.5 m), followed by a stationary phase between 0.5 m and 3 m; values varied between 40% and 45%. The lowest values (<40%) were recorded over 3 m (Table 3). The algae cultivated in the BL has the higher sulphate degree than those cultivated in the BB (6 ± 2.00% vs 8.80 ± 0.86%) (Table 3). Furthermore, in the BL the highest sulphate degree (10%) was attained over 3 m, and the lowest (7.50%) at 2.50 m. Nevertheless, in the BB, the lowest sulphate degree (3.40%) recorded at 3.25 m (Table 3).

The site and the depth does not affect the protein content of the alga (p > 0.05). Accordingly, the contents recorded were 22.02 ± 6.34% and 20.74 ± 7.22% for the BL and the BB respectively (Table 4). The R-PE contents in the BL (0.33 ± 0.12 mg.g^−1^ fwt) was lower (p < 0.05) than that obtained in the BB (0.86 ± 0.31 mg. g^−1^ fwt) and values were homogeneously distributed across the depths in both sites (Table 3).

## Discussion

### The physicochemical parametres of culture site

The lower Secchi depth (2 m) obtained in the BL is due to the turbidity. However, the muddy bottom and the wind increase the turbidity of the water. Likewise, the lagoon receives several urban and industrial discharges from the around cities, other than the sediments from the rivers^24^. Unlike the BL, Secchi depth obtained in the BB occurs in the water depths greater than 20 m as indicated by^25^. The salinity value in the BL is similar to that recorded by^26,27^. The water temperature values reported in our study were similar to that obtained by^26–28^, which were (15–23 °C), (15–25 °C) and (19–20 °C) respectively. In the BL, there is no vertical gradient of salinity or temperature as indicated for other lagoons^29,30^, probably due to the shallow depth. The salinity and temperature values recorded in the BB between March and May are consistent with those reported previously (19 °C and 37 psu)[84].

The nutrient concentrations in the BL are comparable to those of a Mediterranean Sea Lagoon, Lake Burullus in Egypt^31^. Accordingly, the authors reported nutrient concentrations in spring as follows: nitrate (7.41–28.90 µM), ammonium (7.34–34.30 µM) and phosphates (8.59–24.30 µM). In addition, a result of a physicochemical assessment of the Nador Lagoon water quality (Northeast Morocco), indicates a varied nitrate (1.08–29.28 µM) and phosphates (1–7 µM) concentrations^32^. Furthermore, a nitrate (0.35–52.4 µM) and phosphate (0.41–2.24 µM) concentrations were determined in Mar Chiquita, a coastal lagoon in Argentina (South Atlantic Ocean)^33^. According to^34^, there is no vertical stratification of different nutrients (ammonium, nitrate and orthophosphate) in the BL. The lagoon receives in winter and spring an important flow of nutrients (liquid and solid) from their watershed, containing a very high quantity of nitrate compared to the BB^35^. In conclusion, during the culture period, the BB has a higher transparency but a lower nitrogen (nitrate + ammonium) concentration compared to the BL.

### Growth

For *G. gracilis* growth, the DGR recorded in the BL and the BB are in the range of those recorded in outdoor culture, in Tunisia or in others regions, which generally varied between 1% and 4% day^−1^ ^8,9,36,37^. In contrast, indoor culture the DGR attained 10% day^−1^ or higher^38^. The difference between the results could be due to the nitrogen concentration of the medium (nitrate + ammonium), which falls within the range of outdoor concentrations found in other studies. However, in the BL and the BB, the nitrogen concentration is too low (<50 µM) to sustain the high seaweed DGR required for biomass production as indoor culture (>1000 µM) due to the highly nitrophilic character of *G. gracilis*. Hence, the higher growth rate in the BL can be attributed to the nitrogen enrichment due to surface run off into the lagoon, which allows the alga to meet their nitrogen requirements compared to the BB. Moreover, the water transparency, act differently on *G. gracilis* growth^39^. Consequently, lower light and higher nitrogen in the BL enhance DGR in contrast to lower nitrogen and higher light in the BB, which reduce algae growth. Hence, the difference between the findings could be due to the significant effect of light and nitrogen interaction on *G. gracilis* growth as indicated by^39^. In addition, the lower DGR (<3%) obtained in the BL and the BB could be due to the temperature and salinity values, which were out of the optimum growth range. Wide temperature (0–35 °C) and salinity (10–40 psu) tolerance of *Gracilaria* spp. has been reported but the optimum growth has been recorded in a restricted ranges (20 °C–28 °C; 25 psu −30 psu)^40–42^. In contrast, the water temperature and salinity values in the BL and the BB, during the experimental period, are outside the optimum range of alga growth (<20 °C, >30 psu) or near to their lower growth limit. Finally, based on the DGR obtained, the plants reached a harvestable size after 90 days in the BL and 110 days in the BB, but it is only of 30 days in Chilika Lake in India^43^. The difference between results may be attributed to the physicochemical characteristics of the water in both sites.

Our data show a markedly difference in DGR of *G. gracilis*, across the depths in both sites. However, light intensity is a relevant factor, which affects algae growth. In the BL, the highest DGR values were obtained in the shallowest depth (<1 m) and the lowest in deeper one (>3 m). Our findings are consistent with those of^44–47^. Consequently, they indicate that the lower DGR of *Gracilaria* spp. obtained is explained by the reduced light intensity due to the high turbidity essentially in eutrophic lagoon. In contrast, the DGR of *G. gracilis* in the BB was not affected in a depth of 4 m, suggesting the availability of enough light quantities in the studying depths. According to^48,49^, *Gracilaria* can grow in the depths between 8 m and 10 m, but over 4 m their growth is largely affected; especially in a turbid environment that do not let light through.

In our study the PTL is low than that recorded in the same lagoon and culture period, using bottom planting methods^50^. The studies on *G. gracilis* farming carried out in the BL, by these methods, revealed that the PTL constituted a detrimental factor^50^. However, algae losses are related to difficulties in inserting tufts on ropes^50^, epiphytism^51^ and associated fauna, which contain zoological groups that use the genus *Gracilaria* for habitat and food^52^. In our study no epiphytes or epifauna associated to *G. gracilis* in both sites, which could explain the lower PTL. The problems of grazing and entanglement by other epiphytes were controlled upto higher level by the lantern net, which covered all sides of the thallus. The maximum loss was recorded at the shallow depths (<1.5 m) due to fragmentation of the thalli into small parts (<1 cm). However, this size facilitates their escape through the mesh of the net in addition to the decomposition of those that remain trapped. The *Gracilaria* culture like any other mariculture activity has an impact on the marine environment. According to^53,54^, there was a decrease in the growth rate of *Zostera japonica* but an increase in the abundance and diversity of invertebrates in the community under the cultivated ropes of *Gracilaria* spp. In the BL, there is no development of seaweeds over 5 m, which avoids any interaction between *Gracilaria* and other species. Lantern nets developed for our study have more advantages than the benthic or the suspended culture methods using thalli inserted on the ropes^50^. However, *G. gracilis* productivity is higher and the PTL is lower. This advantage is essentially linked to the attenuation of wave effects on *G. gracilis* thalli, which depend on the site and the depth. However, near the sea bottom, the wave velocity decrease (<0.2 m s^−1^), whereas near the surface increase (0.38 ms^−1^), which can explain the higher PTL recorded in this part of the water column. In addition, in our study an improvement in attachment to nets covering the hoops (0.5 m^2^) is a mechanism that enables alga to survive the wave action and currents occur in this zone, which result in higher biomass production. In the BL, the marine wave velocity is low (<0.2 m s^−1^), which could explain their little shearing actions on the thallus^24,55^. In conclusion, the lower PLT obtained, the better we need for *G. gracilis* culture success in these regions.

Based on DGR, after one month when we start from a stocking density of 7.5 kg m^−2^, we will reach a final density of 11.5 kg m^-2^, generating a multiplication factor of 1.5. However, this value is relatively higher than that obtained by the benthic culture system used in the BL^50^ but was similar to those obtained by the suspended culture system in the sea, for other *Gracilaria* species. Accordingly, the multiplication factor values obtained were 1.2 for *Gracilaria caudata*^45^; 2 for *Gracilaria chilensis*^56^; 2.03 for *Gracilaria chilensis*^57^; 2.16 for *Gracilaria chilensis*^58^ and 6.36 for *Gracilaria sp*.^47^.

### Agar yields and composition

Agar yields of *G. gracilis* obtained in the BL and the BB were comparable to those previously found for *G. gracilis* and others species, which varied from 20% to 30%^59–61^. In contrast, a lower agar yield of *G. gracilis* was obtained in a shallow part of the BL^9^. However, not all studies agree with this finding due to the difference between species and culture sites. The multiple environmental factors, such as nutrient status, light, salinity and water temperature, could affect agar yield^2,39^. The higher agar yields obtained in the BB, could be due to the high salinity level, light quantity and a lower nitrate concentrations than that recorded in the BL^39^. The culture depth of *G.gracilis* in the BL and the BB didn’t affect the agar yields in contrary to previous findings in the BL by^9^, which indicated a higher agar yields at a depth of 2 m compared to those obtained at 0.5 m. Unlike the previous work, this study was performed in the eastern part of the lagoon, characterized by a higher turbidity (Secchi depth <0.5 m). Consequently, the amount of light is insufficient (<70 µmol m^2^ s^−1^) to interact with other factors and allowing the alga to produce higher agar yields^39,62^. Accordingly, in the BL the lower light effect on *G.gracilis* at a depth over 2 m, which superior to a critical value indicated above, may be alleviated by the interaction of light and other factors. However, when agar yield was affected, interactions between abiotic factors (light, salinity and nitrogen) alleviate the negative impact and maintain the yield similar to that obtain at the shallow depths^39^. In contrast, light amount in the BB, which is independent of the depths, can explain the homogeneity of the agar yields along water column.

Agar composition (sulphate degree and 3,6-AG content) obtained in both sites (the BL and the BB) was consistent with those of^63^, which indicate that higher agar yields was accompanied by higher 3,6-AG content and lower sulphate degree. The ideal structure of agarocolloid is a non-substituted galactan backbone composed of repeating units of (1,3)-linked-D-galactose and (1,4)-linked 3,6-anhydro-α-L-galactose. However, native agarocolloids are generally a mixture of neutral, sulphated, methylated and pyruvated agarose, which influences their rheological properties. According to^64,65^, the gel properties (gel strength, gelling temperature and melting temperature), which are the most important criteria to evaluated agar, are highly dependent on the amount of sulphate groups as well as the 3,6-AG content. Accordingly, the higher the 3,6-AG content and the lower the sulphate degree, the better agar gel strength. However, the quality of agar from the BB (41.36 ± 3.68% of 3,6-AG and 6.00 ± 2.00% of sulphate) approach that of agarose (48% of 3,6-AG and 2% of sulphate). In contrast, the agar obtained in the BL is of lower quality due to the lower percentage of 3,6-AG and higher sulphate degree (23.30 ± 5.40% of 3,6-AG and 8.80 ± 0.86% of sulphate). Similar results were obtained in the BL^9^.

### Proteins and R-PE contents

The protein contents of *G. gracilis* (>25%), are lower than that obtained by^10,66^ but are higher than those obtained by^67,68^, which indicates a value varying between 11% and 20%. The difference between results could be due to the environment factors and extraction methods. Hence, in both sites (the BL and the BB) ammonium is a limiting factor, which affects positively chlorophyll and proteins contents but negatively the carbohydrate^69^. The ammonium concentration in the lagoon and the bay does not meet the *G. gracilis* requirement to produce proteins contents higher than 20%. In addition, the higher proteins contents were obtained at a salinity lower than 30 psu. The similar proteins contents obtained in both sites despite the difference in nitrate concentration and light quantity, were due to the interaction between them^39^. In addition, the differences between the results could be attributed to the extraction methods. However, enzyme-assisted extraction of *G. gracilis* produces a high proteins contents compared to the native method^13^.

The R-PE is the most abundant phycoerythrins in Rhodophyta^70^, which does not exceed 10 mgg^−1^ in *Gracilaria* species^71^. However, their concentrations were inversely proportional to the growth rate and vary considerably with environmental factors; essentially light^39,72^. The lower pigments contents of *G. gracilis* in the BL compared to the BB, can be attributed to their higher growth. In addition, the difference between the results could be due to the amount of quantity of light available. However, the inverse relationship between R-PE contents and light intensity is well established^72–74^. Accordingly, a decrease in light intensity enhances the alga pigments accumulation^75^. Red algae grow under intense light, accumulate few phycobilisomes and lower phycobiliproteins content compared to those grow under a low light intensity^76,77^. However, the Secchi depth in the BB was higher than the depth of *G.gracilis* culture, which indicates that the amount of light is homogeneous. In contrast the homogeneity of R-PE in the BL despite the Secchi depth (<2.5 m) can be attributed to the effects of others factors. For this purpose, light effect on *G.gracilis* R-PE contents depend mainly on nitrogen concentration, salinity and their interaction^39,78,79^. Probably without the interaction mechanisms, there will be differences in the amount of R-PE between the depths.

### Aquaculture and biotechnology relevance of results

We stated that the lagoon was characterized by a higher nitrogen concentration and a lower amount of light. In contrast the opposite was happened in the bay. The red alga *G. gracilis* grow well under higher nitrogen concentrations and light quantity, as in many coasts of Asian countries. Accordingly, the DGR will be limited in our conditions compared to those in India coasts^43^. However, in those countries, with the same period of culture, we obtain two production cycles (between 30 and 45 days each one) and only one in Tunisia (between 90 and 110 days). In addition, in the Asian regions there are two growth periods of this alga; it is feasible to obtain four production cycles per year^43^. Contrarily, in Tunisia and the Mediterranean region in general, there is only one period of growth (spring) and only one development cycle per year^4,8,50^. Accordingly, what we presented as a result allows us to prepare a pilot scale up, which is necessary before starting *Gracilaria* aquaculture in the Mediterranean region to boost their productivity by suspended methods. The pilot scale up can spend two years (one cycle/year) and a socioeconomic study can be started. If we consider this to be our ultimate goal, the results obtained in this study allowed us to gather maximum information’s on the subject and facilitates the starting alga culture in an industrial way in the near future.

In the world, the market price of *Gracilaria* is related to the agar yields and quality. The *Gracilaria gracilis*, is used in several industrials sectors mainly food due to their agar yields. As the agar yields of alga harvested from the BB was higher with better quality, compared to the BL, the total economic value might remain the same. If the physicochemical parameters of the BL and the BB generate a low DGR, which limit the alga quantities, we propose to improve the total economic value of the biomass by the extraction of others interested molecules such as proteins and R-PE. In our study protein content can be compared to other high-protein foods such as soya (30%), beef (25%) or salmon (20%)^80^. In addition, a valuable phytochemical may be co-extracted with proteins such us polyphenols, pigments and enzymes having higher additional values, which may be of interest^13,14,81^. A sequential extraction of agar and other compounds was developed with fresh *G. verrucosa*^14^, but it is possible to use dried algae instead of fresh. Increasing interest is being given in the last decades to *Gracilaria* drying. Consequently, a drying technologies were developed allowing the recovery of proteins, R-PE and agar in sufficient quantity^82–84^.

Tunisian aquaculture sector have highly improved during the last two decades; indeed, the national production passed from 2000 tons in 2000 to 20 000 tons in 2017 and the number of aquaculture societies was ten times higher^85^. The marine fish farming production represents 90% of the total production. However, we have interested to minimize negative environmental impacts of this activity. The integrated aquaculture systems (IMTA) based on integrated culturing of finfish, seaweed and mussels, have to contribute to the sustainability of aquacultures, but their development requires further research to optimize the technique, which depends on the selected seaweed species and the system of fish farming. In IMTA, the red algae *Gracilaria* utilize photosynthesis to convert inorganic nutrients into organic molecules and reduce the negative environmental impact of aquaculture activity. Consequently, based on the results of growth and biochemical composition obtained in the BB, *Gracilaria gracilis* could be used in IMTA systems due to the homogenous growth and chemical composition obtained. Furthermore, it can absorb a higher quantity of nitrogen, under changing abiotic factors, which can be produced by the system^39^.

## Conclusions

Our results highlight that *G. gracilis*, cultivated in the BL and the BB using suspended method, possess active growth and interesting concentrations of chemical components (agar, proteins, and R-PE). However, we obtain a better productivity, a higher tufts recovery and larger exploited surface than the culture on the substrate. Furthermore, no direct interaction between the alga and the flora. The light was the key factor; the water depth acts differently. However, in the BL the DGR was affected over the depth of 1.5 m but in the BB is not affected up to 4 m. The choice of the depth and site culture may depend on the growth and the final use of biomass (agar, proteins and R-PE). With the higher agar yields in the BL and the BB, suspended culture of *G. gracilis* in the BB is much more attractive due to the higher protein content, R-PE amount and the better agar quality.

## Supplementary information

Dataset 1.

## Data Availability

The datasets generated during the current study are available on request to the corresponding author.
