# Supplementary material for: Effect of Lagoon and Sea Water Depth on Gracilaria gracilis Growth and Biochemical Composition in the Northeast of Tunisia
Source: Sci Rep. 2020 Jun 22;10:10014. doi: 10.1038/s41598-020-66003-y (PMC7308321; doi:10.1038/s41598-020-66003-y)
Supplement: Supplementary file 1 — Dataset 1. [file 41598_2020_66003_MOESM1_ESM.doc]

**EFFECT OF LAGOON AND SEA WATER DEPTH ON *GRACILARIA GRACILIS***

**GROWTH AND BIOCHEMICAL COMPOSITION IN THE NORTHEAST OF TUNISIA**

**Fethi Mensi^1*^, Sarra Nasraoui^2^, Saloua Bouguerra^3^, Aziz Ben Ghedifa^1^, Mohamed Chalghaf^4^**

*^1^Institut National des Sciences et Technologies de la Mer, B^3^Aqua laboratory, Centre de recherche de Kheiredine, 29 Rue Général Kheiredine 2015 Le Kram, Tunisie. Tél/Fax: (216) 71 276 121; ^*^Corresponding author email:* [*mensi.fethi@instm.rnrt.tn*](mailto:mensi.fethi@instm.rnrt.tn)

*^2^Institut National Agronomique de Tunisie 43, Avenue Charles Nicolle 1082 -Tunis- Mahrajène Tunisie*

*^3^Facultés des Sciences Mathématiques physiques et Naturelles Campus Universitaire El-Manar, 2092 El Manar Tunis, Tunisie.*

*^4^Institut supérieur de Pèche et d’Aquaculture de Bizerte, BP 15 Errimel 7080 Menzel Jemil Bizerte Tunisie.*


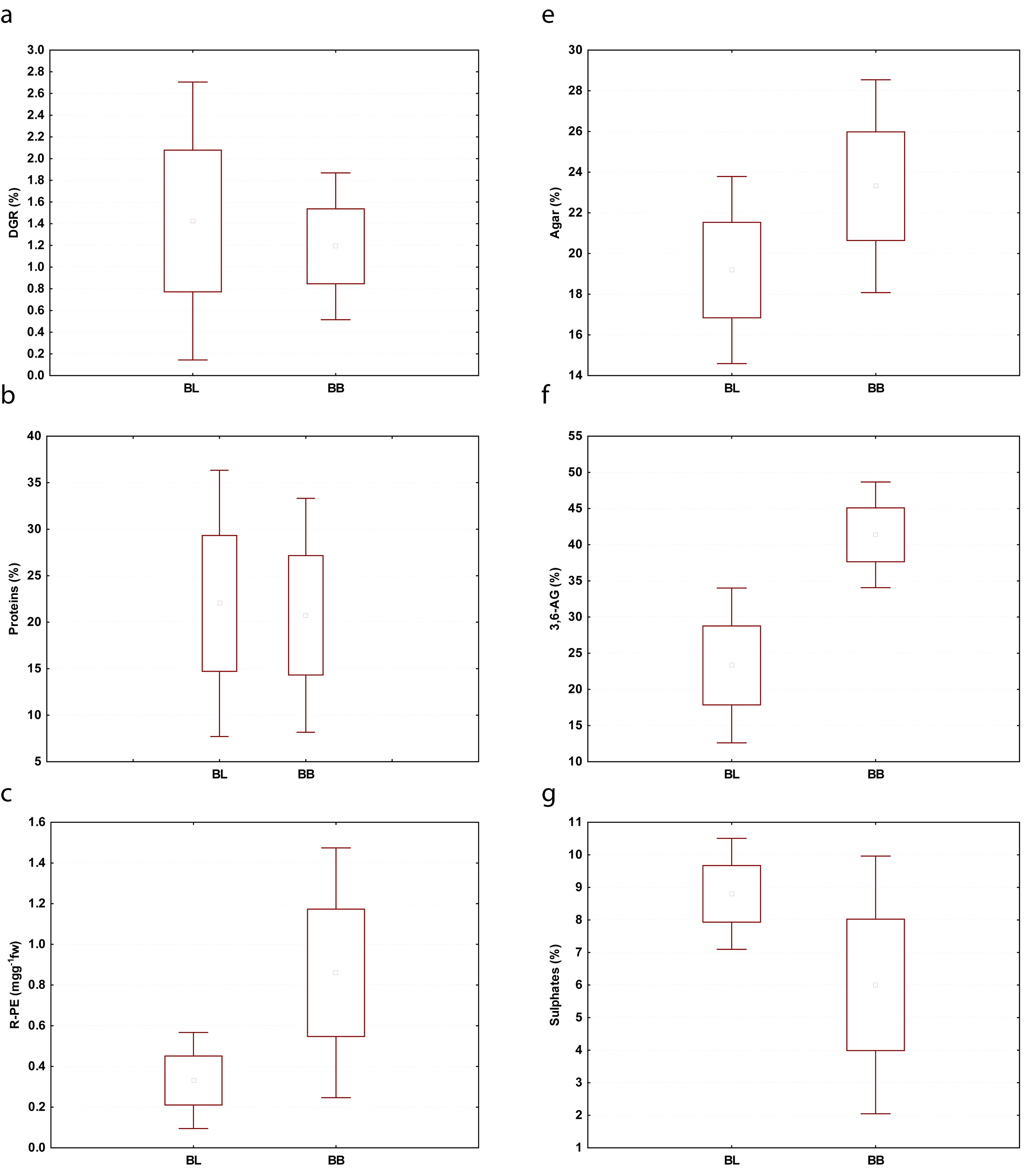


**Supplementary** **Fig. S1** a Box Plot representation of the Growth and proximate biochemical composition of *Gracilaria gracilis* cultivated in Bizerte Lagoon (BL) and Bizerte (BB). (a) Daily growth rate (DGR %); (b) Proteins (%); (c) R-phycoerythrin (R-PE mgg^-1^fw); (e) Agar (%); 3,6 – Anhydrogalactose (3,6-AG %); Sulphates (%).Values are means ± SD of results obtained at different depth (0.25 m to 3.75 m); n = 45.
